# Cyanidin Alleviated CCl_4_-Induced Acute Liver Injury by Regulating the Nrf2 and NF-κB Signaling Pathways

**DOI:** 10.3390/antiox11122383

**Published:** 2022-12-01

**Authors:** Bulei Wang, Shumao Cui, Bingyong Mao, Qiuxiang Zhang, Fengwei Tian, Jianxin Zhao, Xin Tang, Wei Chen

**Affiliations:** 1 State Key Laboratory of Food Science and Technology, Jiangnan University, Wuxi 214122, China; 2 School of Food Science and Technology, Jiangnan University, Wuxi 214122, China; 3 National Engineering Research Center for Functional Food, Jiangnan University, Wuxi 214122, China

**Keywords:** cyanidin, acute liver injury, antioxidation, anti-inflammation, Nrf2 and NF-κB pathway

## Abstract

Acute liver injury has multiple causes and can result in liver failure. In this study, we evaluated the hepatoprotective ability of cyanidin (Cy) and investigated its associated mechanisms. Cy administration significantly and dose-dependently ameliorated acute liver injury induced by carbon tetrachloride (CCl_4_). High-dose Cy showed effects comparable to those achieved by the positive control (silymarin). Severe oxidative stress and inflammatory responses in the liver tissue induced by CCl_4_ were significantly mitigated by Cy supplementation. The total antioxidant capacity and the activity of superoxide dismutase, catalase, and glutathione peroxidase were increased and the content of malondialdehyde, lipid peroxide, tumor necrosis factor α, interleukin-1β, and interleukin-6 were decreased. Additionally, the Nrf2 and NF-κB signaling pathways, which regulate antioxidative and inflammatory responses, were analyzed using quantitative real-time polymerase chain reaction and western blot assay. Cy treatment not only increased Nrf2 transcription and expression but also decreased NF-κB signaling. Moreover, molecular docking simulation indicated that Cy had high affinity for Keap1 and NF-κB/p65, which may promote nuclear translocation of Nrf2 and inhibit that of NF-κB. In summary, Cy treatment exerted antioxidative and anti-inflammatory effects and ameliorated liver injury by increasing Nrf2 and inhibiting the NF-κB pathway, demonstrating the potential of Cy as a therapeutic agent in liver injury.

## 1. Introduction

As a critical internal organ, the liver is responsible for multiple physiological functions, including metabolism, detoxification, and immunity. However, the liver is susceptible to damage induced by exogenous materials, such as alcohol, drugs, and other hepatotoxic chemicals, resulting in liver dysfunction [1,2]. Chemical liver damage, which often aggravates hepatocirrhosis, liver failure, and cancer, is among the most common types of liver injury observed clinically and is a significant burden on the global public health system [3,4]. Currently, many synthetic chemical medicines are widely used to relieve liver injury, although they may cause adverse effects, such as gastrointestinal irritation and weight gain [5]. Therefore, it is imperative to develop novel drugs against liver injury that are more effective and less toxic.

Numerous studies have shown that acute liver injury is often accompanied by excessive oxidative stress and inflammatory responses [6]. The nuclear factor erythroid 2-related factor 2 (Nrf2) and nuclear factor (NF)-κB signaling pathways are the most important signaling pathways involved in regulating inflammation and antioxidation. Nrf2 activation and NF-κB inhibition have been demonstrated to alleviate liver injury. For example, curcumin upregulates the expression of Nrf2 and related downstream antioxidant molecules (superoxide dismutase (SOD), catalase (CAT), heme oxygenase 1 (HO-1), and NAD(P)H quinone dehydrogenase 1 (NQO-1)) to mitigate aflatoxin B1-induced liver injury [7]. Additionally, methoxyeugenol, a molecule extracted from nutmeg and Brazilian red propolis, exhibits hepatoprotective activity both in vitro and in vivo, which may be attributed to the anti-inflammatory effects of targeting the NF-κB signaling pathway [8].

Cyanidin (Cy; 2-(3,4-dihydroxyphenyl) chromenylium-3,5,7-triolis), a naturally occurring flavonoid compound, is found in berries and other fruits [9]. Increasing evidence has suggested that Cy exhibits various bioactivities. Lee et al. demonstrated that Cy exerts strong anticancer activity by significantly inhibiting cellular proliferation and colony formation of colon cancer cells. These anticancer effects are likely associated with suppression of the NF-κB pathway and activation of the Nrf2 pathway [10]. Additionally, Dou et al. reported that Cy exhibits therapeutic potential for preventing osteoclast-related bone disorders. At a low dosage (<1 µg/mL), Cy promotes osteoclastogenesis, whereas at a high dosage (>10 µg/mL), Cy inhibits this process [11]. However, there is very limited information regarding the hepatoprotective activity of Cy, and the mechanism warrants further clarification.

Therefore, in this study, we evaluated the hepatoprotective effects of Cy in carbon tetrachloride (CCl_4_)-induced mice. Moreover, we measured the antioxidant enzyme activity of SOD, CAT, and glutathione peroxidase (GSH-Px), determined the total antioxidant capacity (T-AOC), analyzed the levels of the oxidative products malondialdehyde (MDA) and lipid peroxide (LPO) and detected the contents of the inflammatory factor tumor necrosis factor α (TNF-α), interleukin-1β (IL-1β), and interleukin-6 (IL-6) in Cy-treated mice to assess the antioxidative and anti-inflammatory activity of Cy. Furthermore, the protective mechanism of Cy, including the activation of Nrf2 signaling and inhibition of the NF-κB pathway, was examined using quantitative real-time polymerase chain reaction (qRT-PCR), molecular docking, and western blot analysis. Collectively, our findings indicate that Cy may be a promising therapeutic agent for the safe and effective management of liver injury. 

## 2. Materials and Methods

### 2.1. Materials and Reagents

Cy (>98% purity) was purchased from Chengdu Biopurify Phytochemicals Co., Ltd. (Chengdu, China). Alanine aminotransferase (ALT), aspartate aminotransferase (AST), SOD, CAT, GSH-Px, T-AOC, MDA, and LPO test kits were obtained from Nanjing Jiancheng Bioengineering Institute (Nanjing, China). Alkaline phosphatase (ALP) and BCA protein detection kits were obtained from Beyotime Biotechnology Co., Ltd. (Shanghai, China). Enzyme-linked immunosorbent assay (ELISA) kits for TNF-α, IL-1β, and IL-6, were purchased from SenBeiJia Biological Technology Co., Ltd. (Nanjing, China).

CCl_4_ and other chemical reagents were provided by China National Pharmaceutical Group Co., Ltd. (Beijing, China).

### 2.2. Animals and Experimental Design 

**Scheme 1:**
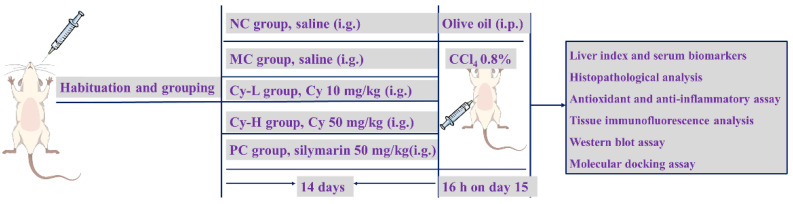
Experimental design.

Specific pathogen-free BALB/c male mice (5–6 weeks old; weighing 18–20 g) were purchased from Gempharmatech Co., Ltd. (Nanjing, China). All mice were allowed free access to commercial food and sterile water. After acclimation for 7 days, 40 mice were randomly divided into five groups (n = 8): (A) normal control (NC), in which the animals were gavaged with physiological saline; (B) model control (MC), in which the animals were gavaged with physiological saline; (C) Cy-L treatment group, in which the animals were gavaged with Cy (10 mg/kg); (D) Cy-H treatment group, in which the animals were gavaged with Cy (50 mg/kg); and (E) positive control (PC), in which the animals were gavaged with silymarin (50 mg/kg). After pre-treatment with Cy and silymarin for 14 days, all mice, except for those in the control group (which were administered an equivalent volume of olive oil), were injected intraperitoneally with 0.8% CCl_4_ (10 mL/kg) in olive oil to induce acute liver injury (Scheme 1). After 16 h, the mice were sacrificed, and blood samples from the retro-orbital sinus of the mice were collected in heparin-free tubes and placed at room temperature for 1 h. The livers were weighed and separated into two parts. One portion was cryopreserved in liquid nitrogen for further analysis, and the other was fixed in 4% formalin for histological observation.

### 2.3. Determination of Serum Liver Function Indices

Blood samples were centrifuged (3500 rpm/min, 10 min) to obtain the sera at 4 °C. The levels of AST, ALT, and ALP were determined using commercial assay kits according to the manufacturer’s instructions. The contents of TNF-α and IL-6 were measured using corresponding ELISA kits.

### 2.4. In Vivo Histopathological Assessment

The fixed liver tissues were dehydrated, embedded in paraffin, and sectioned at a thickness of 4 µm. These samples were then stained with hematoxylin and eosin (H&E) staining and photographed using an optical microscope [12].

Apoptosis in liver tissue was further evaluated using terminal deoxynucleotidyl transferase-mediated dUTP nick-end labeling (TUNEL) assay. Liver sections (4 µm) were dewaxed using water and stained using a TUNEL apoptosis detection kit according to the manufacturer’s instructions (Wuhan Servicebio Technology Co., Ltd., Wuhan, China). TUNEL-positive cells (green) were detected using a fluorescence microscope [12]. The apoptotic index (AI) was also calculated as the number of apoptotic cells.

### 2.5. Assessment of Antioxidant Activity and Inflammatory Response in the Liver 

Samples of liver tissue (0.1 g) were homogenized in cold normal saline (1 mL) and then centrifuged (12,000 rpm/min, 15 min) at 4 °C to collect the tissue supernatant. The protein content was measured using the BCA method. The levels of oxidative stress were assessed by analyzing the antioxidant enzyme activities of SOD, CAT, and GSH-Px, determining the T-AOC, and measuring the levels of the oxidative products MDA and LPO, using corresponding kits. Additionally, the levels of inflammatory cytokines (TNF-α, IL-6, and IL-1β) were measured using corresponding ELISA kits.

### 2.6. qRT-PCR Assay

Changes in the mRNA levels of each gene were quantified using qRT-PCR [13]. Briefly, total hepatic RNA was extracted using TRIzol reagent, and the purity and concentration of the RNA were determined using a NanoDrop 2000C spectrophotometer (Thermo Scientific, Waltham, MA USA). cDNA samples were synthesized using a commercial kit (Vazyme R302-01, Nanjing, China) and further analyzed by qPCR amplification. The sequences of gene-specific primers are listed in Table 1. The relative expression of target genes was determined using the 2^−ΔΔCt^ method, with glyceraldehyde-3-phosphate dehydrogenase (GAPDH) used as an internal reference.

### 2.7. Tissue Immunofluorescence Staining

Immunofluorescence staining of the liver was performed to evaluate the levels of TNF-α and IL-6 (Wuhan Servicebio Technology Co., Ltd., Wuhan, China), and the nuclei were stained with 4′,6-diamidino-2-phenylindole (DAPI). The stained sections were observed and photographed using a fluorescence microscope. 

### 2.8. Western Blotting

Proteins were extracted form liver segments in each group through RIPA lysis, quantified using a BCA assay kit, and denatured with loading buffer in a boiling water bath. Then, the results were separated by SDS-PAGE and transferred to PVDF membranes. After incubation with primary antibodies Nrf2 (1:1000), NF-κB/p65 (1:2000), p-NF-κB/p65 (1:2000), GAPDH (1:2000) and LaminB1 (1:2000) overnight at 4 °C, the membranes were washed with TBST and incubated with horseradish peroxidase labeled secondary antibodies (1:5000). The protein bands were detected using ECL reagents. 

### 2.9. Molecular Docking Analysis

Molecular docking experiments were performed to predict the binding mode between small-molecule ligands and biological macromolecules using AutoDock 4.2 [14]. The three-dimensional structure of Cy (compound CID: 128861) was obtained from the NCBI PubChem database (https://pubchem.ncbi.nlm.nih.gov/, accessed on 9 March 2022). Those of the target proteins, including NF-κB (PDB code: 1IKN) and Keap1 (PDB code: 4L7B), were downloaded from the RCSB Protein Data Bank (http://www.rcsb.org/, accessed on 9 March 2022). For docking, the ligands were prepared by adding all hydrogen atoms and defining rotatable bonds, and target proteins were applied by extracting the primary ligand, removing water molecules, and adding all hydrogen atoms. The exhaustiveness value was set to 20 to increase the docking accuracy. The best-scoring pose, as determined from the binding energy, was selected, and visually analyzed using PyMOL 2.3.0.

### 2.10. Statistical Analysis

All data are represented as individual data points and as the mean ± standard error of the mean. Statistical analysis was performed using SPSS software (version 23.0), and one-way analysis of variance with Tukey’s post hoc test was used for comparisons among multiple groups. All statistical graphs were drawn using Origin software version 2018. * *p* < 0.05 and ** *p* < 0.01 were considered the thresholds for significance.

## 3. Results

### 3.1. Cy Treatment Alleviated CCl_4_-Induced Liver Damage in Mice 

**Figure 1:**
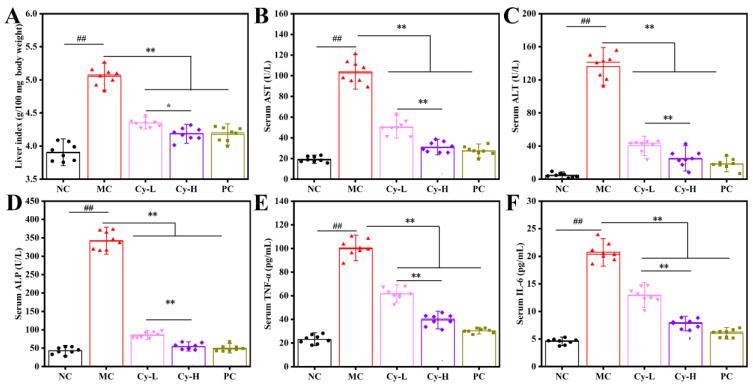
Cy treatment ameliorated CC_4_-induced liver injury mice. (**A**) Liver index. Serum levels of (**B**) AST, (**C**) ALT, (**D**) ALP, (**E**) TNF-α and (**F**) IL-6. ^##^ *p* < 0.01 compared with NC group, * *p* < 0.05 and ** *p* < 0.01 compared with the MC group.

The liver indices and levels of serum biomarkers (ALP, AST and ALP) in the mice are shown in Figure 1A–D. Compared to those in the NC group, the levels of these indicators were significantly increased in the MC group, suggesting that acute liver damage was successfully induced by CCl_4_. However, preventive administration of Cy remarkably and dose-dependently attenuated the increases in biomarker levels in the MC group. Specifically, in the Cy-H group, compared with that in the MC group, the liver index decreased by 17.1%, and the serum levels of AST, ALT, and ALP decreased by 70.0%, 81.5%, and 84.0%, respectively. These reductions did not significantly differ from those in the PC group (17.4%, 73.6%, 86.4%, and 85.5%, respectively). Additionally, the levels of serum TNF-α and IL-6 were used to evaluate the inflammatory response (Figure 1E,F). Injection with CCl_4_ induced serious inflammation, characterized by increased levels of inflammatory factors, and these changes were attenuated by pretreatment with Cy. These results indicate that Cy exerted a hepatoprotective effect against CCl_4_-induced liver injury in vivo.

### 3.2. H&E and TUNEL Staining

**Figure 2:**
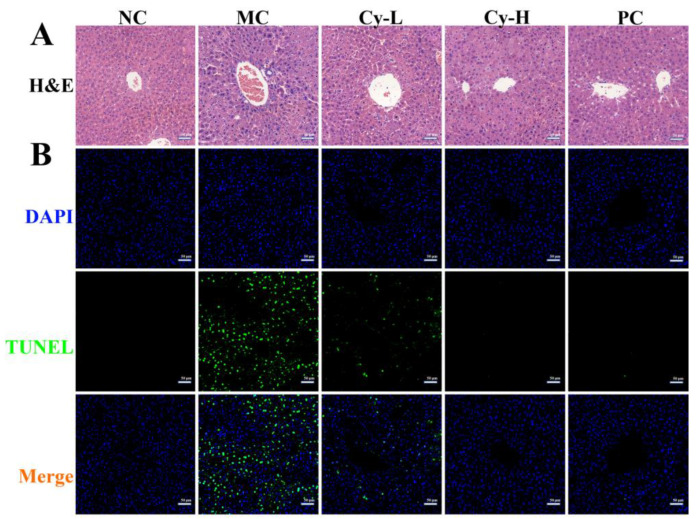
Cy treatment improved hepatic pathological changes in CC_4_-induced liver injury mice. The representative images of (**A**) H&E and (**B**) TUNEL immunofluorescence staining. Scale bars: 50 μm.

The results of liver H&E staining are shown in Figure 2A. No visible histological abnormalities (clear hepatic cells and central veins) were observed in the NC group. In contrast, serious injury, including the disruption of the tissue architecture, inflammatory cell infiltration, and hemorrhage, was observed in the MC group. However, these pathological disorders can be effectively ameliorated by Cy pretreatment. Notably, in the Cy-H group, we observed mitigation of necrosis and reduced inflammatory cell infiltration in a manner comparable to that observed in the PC group, indicating that Cy-H prevented CCl_4_ intoxication. 

Furthermore, cellular apoptosis in the liver tissue was detected using the TUNEL staining assay, in which cell nuclei exhibited blue fluorescence and fragmented DNA exhibited green fluorescence [15]. As presented in Figure 2B, CCl_4_ stress exposure resulted in an increased number of TUNEL-positive cells in the liver tissue compared to that in the NC group. In the Cy-L, Cy-H, and PC groups, the green fluorescence was less intense, suggesting that cell apoptosis was decreased in the liver tissue, which is consistent with the results of H&E staining. The values of AI were 1.4%, 47.8%, 18.9%, 3.5% and 4.3% in NC, MC, Cy-L, Cy-H and PC groups, respectively. Interestingly, the AI did not significantly differ between the Cy-H and PC groups. The pathological images indicated that Cy ameliorated CCl_4_-induced liver injury in a concentration-dependent manner.

### 3.3. Cy Treatment Decreased Hepatic Oxidative Stress in Mice

**Figure 3:**
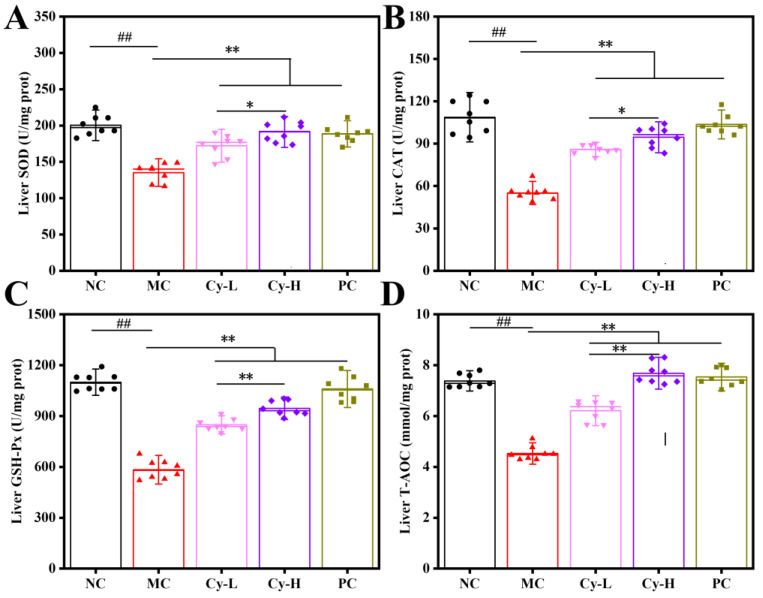
Cy treatment enhanced hepatic antioxidant enzyme activities in CC_4_-induced liver injury mice. The activities of (**A**) SOD, (**B**) CAT, (**C**) GSH-Px, and (**D**) content of T-AOC. ^##^ *p* < 0.01 compared with NC group, * *p* < 0.05 and ** *p* < 0.01 compared with the MC group.

The activity of hepatic antioxidant enzymes (SOD, CAT, and GSH-Px) in the mice is shown in Figure 3A–C. In the MC group, the liver tissues exhibited dramatically lower SOD, CAT, and GSH-Px activity compared to those in the NC group, indicating hepatic cell injury. However, Cy treatment significantly and dose-dependently increased the activity of antioxidant enzymes, compared to those in the NC group. Interestingly, in the Cy-H group, the increases in the SOD, CAT, and GSH-Px activities (41.5%, 71.8%, and 62.3%, respectively) were similar to those in the PC group (39.5%, 88.3%, and 81.8%, respectively), indicating that Cy enhances hepatic antioxidant defense systems by elevating antioxidant activity. Additionally, the T-AOC reflects the compensatory ability of both enzymatic and nonenzymatic antioxidants to respond to external stimuli and maintain bodily functions [16]. As displayed in Figure 3D, the MC group exhibited a lower T-AOC value than that in the NC group. Supplementation with Cy-L and Cy-H significantly increased the hepatic T-AOC by 37.2% and 69.7%, respectively, compared with that in the MC group.

**Figure 4:**
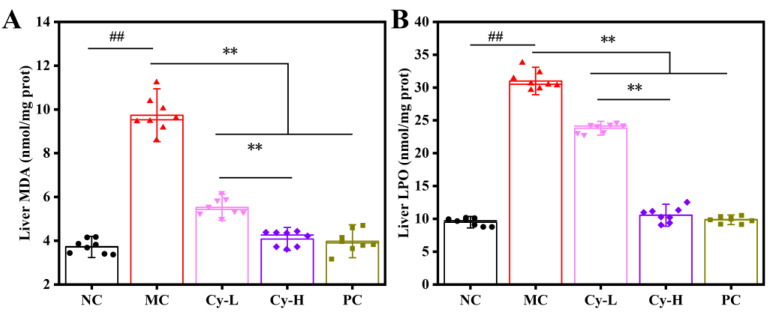
Cy treatment decreased the hepatic levels of oxidative products in CC_4_-induced liver injury mice. The contents of (**A**) MDA and (**B**) LPO. ^##^ *p* < 0.01 compared with NC group, ** *p* < 0.01 compared with the MC group.

The hepatic levels of the oxidative products MDA and LPO in the five groups are shown in Figure 4A,B. Intraperitoneal injection with CCl_4_ caused an approximately threefold increase in the levels of MDA (from 3.7 to 9.5 nmol/mg) and LPO (from 9.5 to 31.0 nmol/mg) compared to those in the NC group, indicating the presence of oxidative damage. Conversely, Cy pretreatment prevented abnormal increases in the levels of the oxidation products observed in the MC group. In the Cy-L and Cy-H groups, the MDA levels decreased significantly by 4.2 and 5.6 nmol/mg, respectively, and LPO levels decreased by 7.1 and 20.4 nmol/mg, respectively. The contents of oxidative products in the Cy-H group were not significantly different from those in the NC group.

### 3.4. Cy Treatment Decreased Hepatic Inflammatory Responses in Mice 

**Figure 5:**
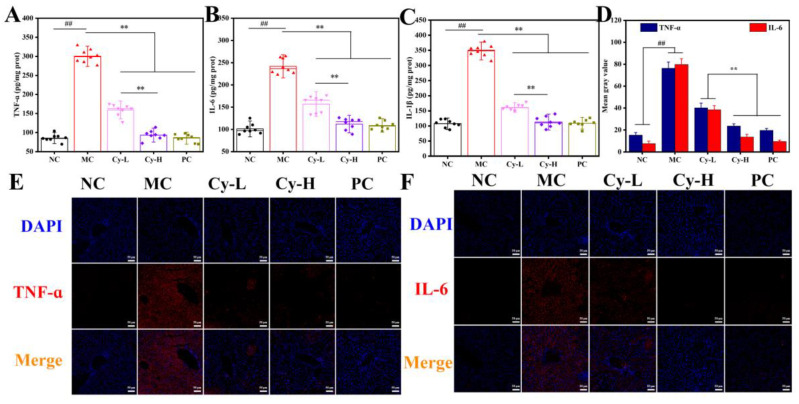
Cy treatment inhibited hepatic inflammatory responses in CC4-induced liver injury mice. The levels of (**A**) TNF-α, (**B**) IL-6, (**C**) IL-1β, (**D**) gray value analysis of cytokine expression and representative images of immunofluorescence staining for (**E**) TNF-α and (**F**) IL-6. Scale bars: 50 μm. ^##^ *p* < 0.01 compared with NC group, ** *p* < 0.01 compared with the MC group.

TNF-α, IL-6, and IL-1β are crucial cytokines that mediate inflammatory responses. As shown in Figure 5A–C, compared with the NC group, CCl_4_ administration resulted in significant inflammation, as demonstrated by the increased levels of hepatic pro-inflammatory mediators. However, compared with the MC group, in which the levels of TNF-α, IL-6, IL-1β were 300.3, 241.9, and 241.9 pg/mg, respectively, the groups pretreated with Cy-L and Cy-H exhibited significantly lower levels of TNF-α (159.0 and 93.3 pg/mg, respectively), IL-6 (157.1 and 112.0 pg/mg, respectively), and IL-1β (161.2 and 112.8 pg/mg, respectively). Interestingly, in the Cy-H group, the levels of hepatic TNF-α, IL-6, and IL-1β were significantly decreased (by 68.9%, 53.7%, and 67.5%, respectively) compared those in the MC group. The decreases observed following Cy-H treatment were slightly lesser in magnitude than those induced by treatment with PC (71.5%, 55.0%, and 68.8%, respectively).

The levels of TNF-α and IL-6 were further evaluated by tissue immunofluorescence staining analysis, in which blue fluorescence represents the nuclei and red fluorescence indicates inflammatory factors. As shown in Figure 5E, F, the strong fluorescence signals for TNF-α (**E**) and IL-6 (**F**) in the red channel suggested an excessive inflammatory response in the MC group. Conversely, pretreatment with Cy and silymarin reduced the intensity of red fluorescence, indicating a decrease in inflammatory cytokine levels, which agreed with the results of ELISA. These in vivo findings suggest that Cy mitigated CCl_4_-induced oxidative stress and inflammation, and this specific mechanism will be investigated in the next section.

### 3.5. Cy Treatment Activated the Nrf2 Signaling Pathway and Inhibited the NF-κB Signaling Pathway

**Figure 6:**
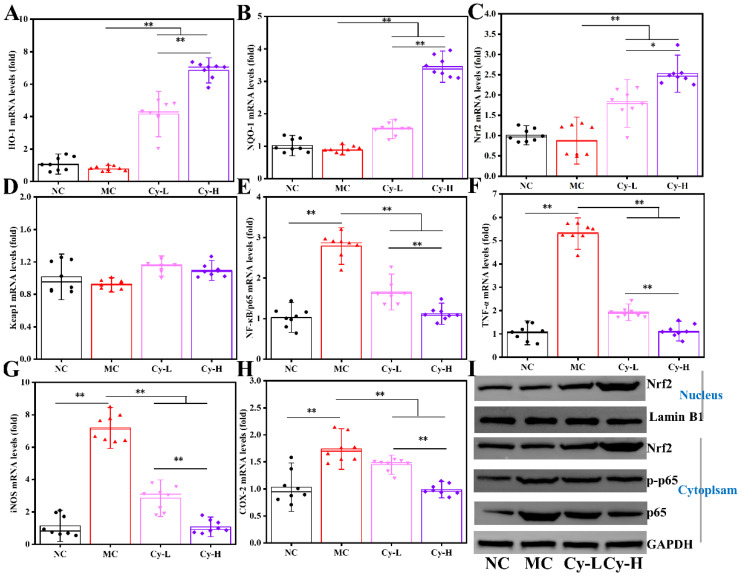
Cy treatment enhanced hepatic Nrf2 and inhibited the NF-κB pathway. mRNA level changes in (**A**) HO-1, (**B**) NQO-1, (**C**) Nrf2, (**D**) Keap1, (**E**) NF-κB/p65, (**F**) TNF-α, (**G**) iNOS, and (**H**) COX-2, and (**I**) western blot analysis of protein expression (Nrf2, p65 and p-p65). * *p* < 0.05 and ** *p* < 0.01 compared with the MC group.

The transcription of genes in the Nrf2 antioxidant pathway (Nrf2, Keap1, HO-1, and NQO-1) in the liver tissue was determined using qRT-PCR (Figure 6A–D). Compared with those in the NC group, the relative mRNA levels of HO-1 and NQO-1 in the MC group were significantly decreased by 28.1% and 13.0%, respectively. This downregulation was reversed by Cy in a concentration-dependent manner. In the Cy-L and Cy-H groups, the mRNA levels of HO-1 were dramatically increased (438.9% and 788.1%, respectively) and those of NQO-1 were enhanced (72.6% and 289.8%, respectively), compared with those in the MC group. Moreover, the mRNA transcription of Nrf2, the upstream regulator of HO-1 and NQO-1, was significantly improved (103.6% and 187.4%, respectively) in the Cy-L and Cy-H groups, compared with that in the MC group. In contrast, there was no remarkable difference in the transcriptional levels of Keap1, although the Cy group exhibited a slight enhancement, compared to in the MC group. Additionally, increased in Nrf2 expression and nuclear translocation were confirmed by western blot analysis (Figure 6I). The expression of Nrf2 in nucleocytoplasmic separation by western blotting further confirmed that Cy treatment promoted Nrf2 translocation to the nucleus, resulting in increased antioxidant activity.

The relative mRNA levels of genes in the NF-κB inflammatory pathway (NF-κB/p65, TNF-α, iNOS, and COX-2) in the liver tissue were quantified, as shown in Figure 6E–H. The MC group exhibited higher levels of NF-κB/p65, TNF-α, iNOS, and COX-2 transcription than those in the NC group, indicating intense inflammation. In contrast, pretreatment with Cy significantly downregulated the mRNA expression of NF-κB/p65 and its corresponding target genes, compared with that in the MC group. In particular, in the Cy-H group, the mRNA level of NF-κB/p65 decreased by 59.9%, and downstream target genes (TNF-α, iNOS, and COX-2) were downregulated by 78.8%, 85.0%, and 43.2%, respectively, compared with those in the MC group. The decreased NF-κB/p65 levels were verified by western blot assay (Figure 6I). The expression of p65 and p-p65 was clearly down-regulated following Cy treatment compared to that in the MC group. These findings indicated that Cy ameliorated the CCl_4_-induced inflammatory response by suppressing the NF-κB signaling pathway.

**Figure 7:**
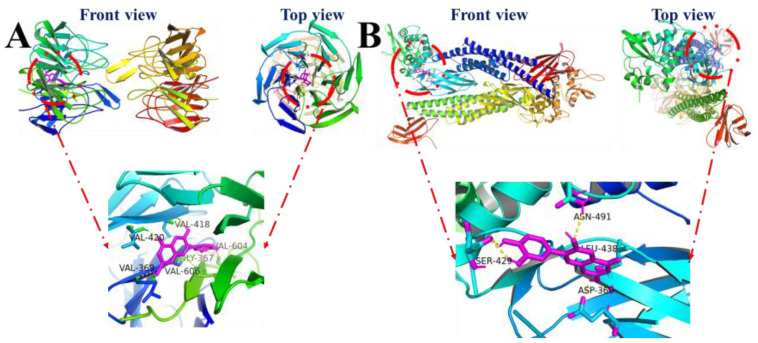
Theoretical binding modes between (**A**) Cy and Keap1, and (**B**) Cy and NF-κB/p65.

Molecular docking studies are considered a powerful tool for predicting the potential targets of bioactive molecules [17]. The theoretical binding modes of Cy with its target proteins (Keap1 and NF-κB) are shown in Figure 7A,B. The ligand of Cy exhibited strong affinity for Keap1 and NF-κB/p65. Keap1 combines with Nrf2 and regulates the cytoplasmic-nuclear shuttling of Nrf2. The molecular docking results suggested that Cy anchors in Keap1 to form a complex through hydrogen bonds with various residues (Gly367, Val369, Val418, Val420, Val604, and Val606). The total interaction energy was −27.949 kJ/mol, indicating the stability of the complex. Thus, we speculated that Cy may occupy the binding site of Keap1, potentially interfering with the association between Nrf2 and Keap1; as a result, free Nrf2 may be transferred to the cell nucleus. Similarly, Cy bound to NF-κB/p65, via Asp369, Ser429, Leu438 and Asn491, with a total interaction energy of −13.43 kJ/mol. This interaction may inhibit NF-κB nuclear translocation and inactivate the NF-κB pathway, thereby suppressing inflammation.

## 4. Discussion

We analyzed the hepatoprotective activity of Cy against CCl_4_-induced toxicity in mice and elucidated its possible mechanism of action. CCl_4_, an acknowledged hepatotoxin, can attack and damage hepatocytes. CCl_4_ has been widely employed to establish experimental models of hepatic injury for developing potential treatment strategies [18]. Hepatic ALT and ALP are located in the cytoplasm, whereas AST is mainly distributed in the mitochondria. After liver cell damage, AST, ALT, and ALP leak into the bloodstream as a result of the increase in hepatocellular membrane permeability. Thus, serum AST, ALT, and ALP are recognized as biomarkers for liver injury [19]. Moreover, the liver index is considered an important indicator for evaluating the degree of experimental liver damage [20]. In this study, intraperitoneal injection of CCl_4_ caused a remarkable increase in the liver index and in the serum levels of AST, ALT, and ALP, indicating serious liver injury. However, supplementation with different doses of Cy mitigated these increases. In particular, high-concentration Cy had similar effects to the positive control, suggesting that Cy can restore liver function by enhancing cytomembrane stability. The results of pathological observations (H&E and TUNEL staining) corroborated these findings. 

Numerous studies have indicated that oxidative stress and inflammatory responses are the main pathogenic factors in CCl_4_-induced liver injury [21,22]. In brief, when CCl_4_ enters hepatocytes, it is metabolized by the cytochrome 450 enzyme family (CYP family), leading to the generation of the trichloromethyl radical (CCl_3_^•^) and the trichloromethyl peroxide radical (Cl_3_COO^•^). These free radicals, exhibit high reactivity and instability and attack proteins and lipids causing lipid peroxidation and decreasing the levels of cell membrane proteins. The radicals also impair the antioxidant defense system in vivo by depleting reducing agents and inhibiting antioxidant enzymes production. These effects contribute to oxidative stress and induce cell damage and death [23]. Oxidative stress also triggers activation of the NF-κB signaling pathway, resulting in neutrophil infiltration and subsequent inflammatory injury to the liver [24]. 

Normally, reactive oxygen species (ROS) in organisms are maintained at physiological levels by antioxidant enzymes and non-enzymatic antioxidants in vivo. As important antioxidant enzymes, SOD catalyzes the formation of hydrogen peroxide (H_2_O_2_) from superoxide radicals, and CAT further degrades H_2_O_2_ into water. GSH-Px also plays a role in eliminating peroxides and hydroxyl radicals [25]. Additionally, non-enzymatic antioxidants, such as GSH, vitamins, and ubiquinone, directly scavenge ROS, which can be indirectly reflected by the T-AOC [26]. Moreover, MDA and LPO are products of lipid peroxidation [27]. Thus, SOD, CAT, GSH-Px, T-AOC, MDA, and LPO are commonly used as biomarkers to assess oxidative stress. Our results demonstrated that CCl_4_-induced oxidative damage in the liver resulted in decreased antioxidant enzyme activity and increased oxidant production. However, Cy administration restored the antioxidant system in a dose-dependent manner, protecting the liver tissue against CCl_4_-mediated oxidative stress.

Previous studies have suggested that inflammation plays an important role in the pathogenesis and progression of CCl_4_-induced liver damage [28]. Kupffer cells, as resident hepatic macrophages, are activated by oxidative stress to release inflammatory factors (TNF-α, IL-6, and IL-1β) that aggravate liver injury. TNF-α promotes the hepatic migration of neutrophils and monocytes, exacerbating hepatocyte injury via overproduction of proteolytic enzymes and ROS [29]. IL-6 and IL-1β activate T lymphocytes and lymphocytes, respectively, to accelerate the inflammatory response [30]. Our results indicate that treatment with Cy significantly downregulated the hepatic levels of pro-inflammatory cytokines to alleviate CCl_4_-induced inflammation. 

As discussed above, the hepatoprotective effects of Cy are closely related to its antioxidant and anti-inflammatory properties. Thus, the effects of Cy on the Nrf2 and NF-κB signaling pathways in the liver were evaluated. The Nrf2 pathway is a well-known central orchestrator of redox homeostasis. Under normal conditions, Nrf2 is ubiquitinated and degraded in the cytoplasm by binding to Keap1. However, under stress conditions, Nrf2 dissociates from Keap1 and translocates into the nucleus to bind to the antioxidant response element, which initiates transcription and expression of downstream genes, such as HO-1, NQO-1, SOD, and CAT, which contribute to resistance to oxidative stress [31,32]. In this experiment, we found that HO-1 and NQO-1, two critical antioxidant enzymes, were remarkably upregulated at the transcriptional level by supplementation with Cy, compared to those in the model group. Furthermore, Cy treatment increased the mRNA and protein levels of the upstream regulator Nrf2 in a dose-dependent manner. The molecular docking study revealed that Cy exhibited good affinity for Keap1 through hydrogen bonding, which may interfere with formation of the Nrf2-Keap1 complex. This frees Nrf2 and accelerates Nrf2 nuclear translocation, thereby activating the Nrf2 signaling pathway. A similar result was reported previously; dietary supplementation with *Eucommia ulmoides* leaf extract upregulated the mRNA expression levels of Nrf2, CAT, and SOD, promoting the intestinal antioxidant capacity and improving the disease resistance of *Oreochromis niloticus* against *Streptococcus agalactiae* [33]. Therefore, additional investigations, such as co-immunoprecipitation and capillary electrophoresis, should be conducted to clarify the direct targets of Cy. 

NF-κB, a pivotal regulator of inflammation, usually resides in the cytoplasm in an inactive state. When cells receive inflammatory signals, NF-κB is activated and it translocates into the nucleus, upregulating the expression of pro-inflammatory cytokines (TNF-α, IL-1α, IL-1β, IL-6, COX-2, and iNOS) and initiating inflammatory injury [34]. Our experiments confirmed that Cy administration significantly downregulated the mRNA and protein levels of NF-κB and its downstream genes (TNF-α, COX-2, and iNOS). Moreover, the results of molecular modeling predicted that Cy may bind to NF-κB/p65, inhibiting its nuclear localization. These experiments indicated that Cy weakened NF-κB signaling and suppressed the CCl_4_-induced inflammatory response. These findings were consistent with those of a study showing that *Salvia hispanica* L. (chia) seed decreases NF-κB/p65 expression and plasma TNF-α levels and attenuates the metabolic syndrome induced by a sucrose-rich diet [35]. 

## 5. Conclusions

**Scheme 2:**
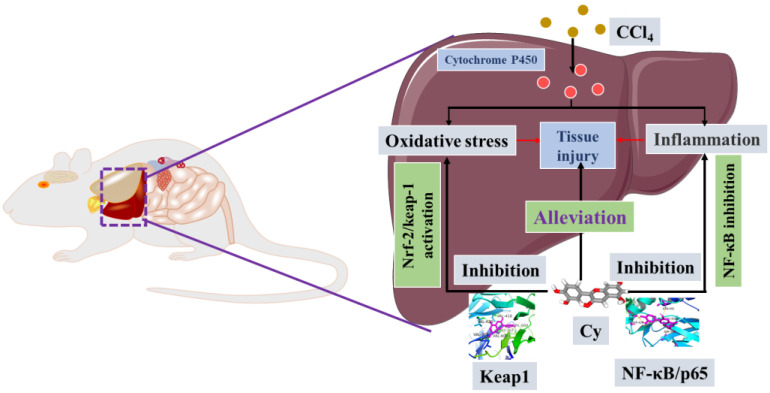
Potential mechanism of Cy-mediated amelioration of CCl_4_-induced liver injury.

Liver diseases are associated with high morbidity and mortality rates worldwide. Our findings suggest that Cy ameliorated CCl_4_-induced liver injury through antioxidative and anti-inflammatory mechanisms. Specifically, Cy enhanced the activity of antioxidant enzymes (SOD, CAT, and GSH-Px), increased the T-AOC, decreased the levels of oxidative products (MDA and LPO), and decreased the levels of pro-inflammatory cytokines (TNF-α, IL-β, and IL-6). Additionally, Cy, as an inhibitor of Keap1 and NF-κB, enhanced Nrf2 pathway activity by increasing Nrf2 transcription and promoting Nrf2 nuclear translocation, while suppressing NF-κB pathway activity by decreasing NF-κB/p65 expression and inhibiting NF-κB nuclear translocation (Scheme 2). 

## Figures and Tables

**Table 1:** Primer sequences used for qRT-PCR analysis.

Genes	Primer Sequences (5′→3′)
HO-1	Forward: CGTGCTCGAATGAACACTCT Reverse: GGAAGCTGAGAGTGAGGACC
NQO-1	Forward: CAGCCAATCAGCGTTCGGTA Reverse: TTGCTGTTGAGGTCGCAGGAG
Nrf2	Forward: CAGCCATGACTGATTTAAGCAG Reverse: CAGCTGCTTGTTTTCGGTATTA
Keap1	Forward: GACTGGGTCAAATACGACTGC Reverse: GAATATCTGCACCAGGTAGTCC
NF-κB/p65	Forward: AGGCTTCTGGGCCTTATGTG Reverse: TGCTTCTCTCGCCAGGAATAC
TNF-α	Forward: ATGTCTCAGCCTCTTCTCATTC Reverse: GCTTGTCACTCGAATTTTGAGA
iNOS	Forward: AGACTGGATTTGGCTGGTCCCTCC Reverse: AGAACTGAGGGTACATGCTGGAGCC
COX-2	Forward: GGGTGTCCCTTCACTTCTTTCA Reverse: COX-2 TGGGAGGCACTTGCATTGA
GAPDH	Forward: GGTGAAGGTCGGTGTGAACG Reverse: CCCGTAGGGCGATTACAGTC

## Data Availability

All data is contained within the article.

## References

[1] Meng X., Tang G.-Y., Liu P.-H., Zhao C.-J., Liu Q., Li H.-B. (2020). Antioxidant activity and hepatoprotective effect of 10 medicinal herbs on CCl_4_-induced liver injury in mice. World J. Gastroenterol..

[2] Chang S.N., Kim S.H., Dey D.K., Park S.M., Nasif O., Bajpai V.K., Kang S.C., Lee J., Park J.G. (2021). 5-*O*-Demethylnobiletin alleviates CCl_4_-Induced acute liver injury by equilibrating ROS-mediated apoptosis and autophagy induction. Int. J. Mol. Sci..

[3] Wu Y., He Y., Wang R., Zhao X. (2021). Preventive effect of flavonoid extract from the peel of gonggan (*Citrus reticulata* Blanco Var. Gonggan) on CCl_4_-induced acute liver injury in mice. J. Inflamm. Res..

[4] Xin C., Liu S., Qu H., Wang Z. (2021). The novel nanocomplexes containing deoxycholic acid-grafted chitosan and oleanolic acid displays the hepatoprotective effect against CCl_4_-induced liver injury in vivo. Int. J. Biol. Macromol..

[5] Cheng Y., Xie Y., Ge J.-C., Wang L., Peng D.-Y., Yu N.-J., Zhang Y., Jiang Y.-H., Luo J.-P., Chen W.-D. (2021). Structural characterization and hepatoprotective activity of a galactoglucan from *Poria cocos*. Carbohyd. Polym..

[6] Dai C., Li H., Wang Y., Tang S., Velkov T., Shen J. (2021). Inhibition of oxidative stress and ALOX12 and NF-κB pathways contribute to the protective effect of baicalein on carbon tetrachloride-induced acute liver injury. Antioxidants.

[7] Wang Y., Liu F., Liu M., Zhou X., Wang M., Cao K., Jin S., Shan A., Feng X. (2022). Curcumin mitigates aflatoxin B1-induced liver injury via regulating the NLRP3 inflammasome and Nrf2 signaling pathway. Food Chem. Toxicol..

[8] de Souza Basso B., Haute G.V., Ortega-Ribera M., Luft C., Antunes G.L., Bastos M.S., Carlessi L.P., Levorse V.G., Cassel E., Donadio M.V.F. (2021). Methoxyeugenol deactivates hepatic stellate cells and attenuates liver fibrosis and inflammation through a PPAR-γ and NF-kB mechanism. J. Ethnopharmacol..

[9] Khashkhashi-Moghadam S., Ezazi-Toroghi S., Kamkar-Vatanparast M., Jouyaeian P., Mokaberi P., Yazdyani H., Amiri-Tehranizadeh Z., Saberi M.R., Chamani J. (2022). Novel perspective into the interaction behavior study of the cyanidin with human serum albumin-holo transferrin complex: Spectroscopic, calorimetric and molecular modeling approaches. J. Mol. Liq..

[10] Lee D.-Y., Yun S.-M., Song M.-Y., Jung K., Kim E.-H. (2020). Cyanidin chloride induces apoptosis by inhibiting NF-κB signaling through activation of Nrf2 in colorectal cancer cells. Antioxidants.

[11] Dou C., Li J., Kang F., Cao Z., Yang X., Jiang H., Yang B., Xiang J., Xu J., Dong S. (2016). Dual effect of cyanidin on RANKL-induced differentiation and fusion of osteoclasts. J. Cell. Physiol..

[12] Niu J., Wang S., Wang B., Chen L., Zhao G., Liu S., Wang S., Wang Z. (2020). Structure and anti-tumor activity of a polysaccharide from *Bletilla ochracea* Schltr. Int. J. Biol. Macromol..

[13] He Z., Chen S., Pan T., Li A., Wang K., Lin Z., Liu W., Wang Y., Wang Y. (2022). Ginsenoside Rg2 ameliorating CDAHFD-induced hepatic fibrosis by regulating AKT/mTOR-mediated autophagy. J. Agric. Food Chem..

[14] Morris G.M., Huey R., Lindstrom W., Sanner M.F., Belew R.K., Goodsell D.S., Olson A.J. (2009). AutoDock4 and AutoDockTools4: Automated docking with selective receptor flexibility. J. Comput. Chem..

[15] Zhang J., Zhao Y., Sun N., Song M., Chen Y., Li L., Cui H., Yang H., Wang C., Zhang H. (2022). Lycopene alleviates chronic stress-induced spleen apoptosis and immunosuppression via inhibiting the notch signaling pathway in rats. J. Agric. Food Chem..

[16] Liu F., Qu Y.-K., Geng C., Wang A.-M., Zhang J.-H., Chen K.-J., Liu B., Tian H.-Y., Yang W.-P., Yu Y.-B. (2020). Effects of hesperidin on the growth performance, antioxidant capacity, immune responses and disease resistance of red swamp crayfish (*Procambarus clarkii*). Fish Shellfish Immunol..

[17] Mahdavi A., Mohammadsadeghi N., Mohammadi F., Saadati F., Nikfard S. (2022). Evaluation of inhibitory effects of some novel phenolic derivatives on the mushroom tyrosinase activity: Insights from spectroscopic analyses, molecular docking and in vitro assays. Food Chem..

[18] Yue H., Cai W., Li Y., Feng X., Dong P., Xue C., Wang J. (2021). A novel sialoglycopeptide from *Gadus morhua* eggs prevents liver fibrosis induced by CCl_4_ via downregulating FXR/FGF15 and TLR4/TGF-β/Smad pathways. J. Agric. Food Chem..

[19] Guo W., Xiang Q., Mao B., Tang X., Cui S., Li X., Zhao J., Zhang H., Chen W. (2021). Protective effects of microbiome-derived inosine on lipopolysaccharide-induced acute liver damage and inflammation in mice via mediating the TLR4/NF-κB pathway. J. Agric. Food Chem..

[20] Wang R., Yang Z., Zhang J., Mu J., Zhou X., Zhao X. (2019). Liver injury induced by carbon tetrachloride in mice is prevented by the antioxidant capacity of Anji white tea polyphenols. Antioxidants.

[21] Zhan J., Cao H., Hu T., Shen J., Wang W., Wu P., Yang G., Ho C.-T., Li S. (2021). Efficient preparation of black tea extract (BTE) with the high content of theaflavin mono-and digallates and the protective effects of BTE on CCl_4_-induced rat liver and renal injury. J. Agric. Food Chem..

[22] Bekkouch O., Dalli M., Harnafi M., Touiss I., Mokhtari I., Assri S.E., Harnafi H., Choukri M., Ko S.-J., Kim B. (2022). Ginger (*Zingiber officinale* Roscoe), lemon (*Citrus limon* L.) juices as preventive agents from chronic liver damage induced by CCl_4_: A Biochemical and Histological Study. Antioxidants.

[23] Long X., Wang P., Zhou Y., Wang Q., Ren L., Li Q., Zhao X. (2022). Preventive effect of *Lactobacillus plantarum* HFY15 on carbon tetrachloride (CCl_4_)-induced acute liver injury in mice. J. Food Sci..

[24] Wu Z., Sun L., Chen R., Wen S., Li Q., Lai X., Zhang Z., Cao F., Sun S. (2022). Chinese tea alleviates CCl_4_-induced liver injury through the NF-κB or Nrf2 signaling pathway in C57BL-6J mice. Nutrients.

[25] Li F., Liao X., Jiang L., Zhao J., Wu S., Ming J. (2022). Orientin attenuated-GalN/LPS-induced liver injury through the inhibition of oxidative stress via Nrf2/Keap1 Pathway. J. Agric. Food Chem..

[26] Guo F., Zhuang X., Han M., Lin W. (2020). Polysaccharides from *Enteromorpha prolifera* protect against carbon tetrachloride-induced acute liver injury in mice via activation of Nrf2/HO-1 signaling, and suppression of oxidative stress, inflammation and apoptosis. Food Funct..

[27] Zeng L., Zhou J., Wang X., Zhang Y., Wang M., Su P. (2021). Cadmium attenuates testosterone synthesis by promoting ferroptosis and blocking autophagosome-lysosome fusion. Free Radic. Biol. Med..

[28] Yue H., Wang P., Zhang L., Ning D., Cai W., Wang Y., Wang J. (2021). Sialoglycoproteins isolated from the eggs of *Carassius auratus* alleviates CCl_4_-induced liver injury via downregulation of the IRE-α/NF-κB signaling pathway. J. Food Biochem..

[29] Li Y., Lv L., Ye J., Fang D., Shi D., Wu W., Wang Q., Wu J., Yang L., Bian X. (2019). Bifidobacterium adolescentis CGMCC 15058 alleviates liver injury, enhances the intestinal barrier and modifies the gut microbiota in D-galactosamine-treated rats. Appl. Microbiol. Biotechnol..

[30] Yang K., Zou Z., Wu Y., Hu G. (2020). MiR-195 suppression alleviates apoptosis and oxidative stress in CCl_4_-induced ALI in mice by targeting Pim-1. Exp. Mol. Pathol..

[31] Zhang X., Chen Y., Li H., Chen B., Liu Z., Wu G., Li C., Li R., Cao Y., Zhou J. (2022). Sulforaphane acts through NFE2L2 to prevent hypoxia-induced apoptosis in porcine granulosa cells via activating antioxidant defenses and mitophagy. J. Agric. Food Chem..

[32] Wang B., Tang X., Mao B., Zhang Q., Tian F., Zhao J., Cui S., Chen W. (2022). Anti-aging effects and mechanisms of anthocyanins and their intestinal microflora metabolites. Crit. Rev. Food Sci..

[33] Huang D., Zhu J., Zhang L., Ge X., Ren M., Liang H. (2022). Dietary Supplementation with *Eucommia ulmoides* Leaf extract improved the intestinal antioxidant capacity, immune response, and disease resistance against *Streptococcus agalactiae* in genetically improved farmed tilapia (GIFT.; *Oreochromis niloticus*). Antioxidants.

[34] Dong J., Ping L., Meng Y., Zhang K., Tang H., Liu D., Li B., Huo G. (2022). Bifidobacterium longum BL-10 with Antioxidant Capacity ameliorates lipopolysaccharide-induced acute liver injury in mice by the nuclear factor-κB pathway. J. Agric. Food Chem..

[35] Joubert M.B.V., Ingaramo P., Oliva M.E., D’Alessandro M.E. (2022). *Salvia hispanica* L. (chia) seed ameliorates liver injury and oxidative stress by modulating NrF2 and NF-κB expression. Food Funct..

